# Marinated oven-grilled beef entrecôte meat from a bovine farm: Evaluation of resultant physicochemical and organoleptic attributes

**DOI:** 10.7717/peerj.15116

**Published:** 2023-03-16

**Authors:** Charles Odilichukwu R. Okpala, Szymon Juchniewicz, Katarzyna Leicht, Małgorzata Korzeniowska, Raquel P. F. Guiné

**Affiliations:** 1 UGA Cooperative Extension, College of Agricultural and Environmental Sciences, University of Georgia, Athens, Georgia, United States; 2 Department of Functional Food Products Development, Faculty of Biotechnology and Food Sciences, Wroclaw University of Environmental and Life Sciences, Wroclaw, Poland; 3 Centre for Natural Resources, Environment and Society (CERNAS-IPV), Polytechnic Institute of Viseu, Viseu, Portugal

**Keywords:** Beef entrecôte, Marination, Herbs and spices, Product development, Thermal processing

## Abstract

Understanding the impact that combined action of marination and oven grill processes would have on such meat products as beef entrecôte is crucial from both consumer appeal and product development standpoints. Therefore, different marinated oven-grilled beef entrecôte meat specifically evaluating resultant physicochemical and organoleptic attributes were studied. The beef entrecôte meat was provided by a reputable local bovine farm/slaughter at Wroclaw, Poland. Physicochemical attributes involved antioxidant (2,2′-azinobis(3-ethylbenzothiaziline-6-sulfonate) (ABTS), 2,2-diphenyl-1-picrylhydrazyl (DPPH), ferric reducing antioxidant power (FRAP)), (pH, thiobarbituric acid reactive substance (TBARS), cooking weight loss, L*a*b* color, and textural cutting force). Organoleptic attributes involved sensory (flavour, appearance, tenderness, taste) and texture (hardness, chewiness, gumminess, graininess, and greasiness) aspects. Different marination variants involved constituent 0.5%, 1%, and 1.5% quantities of cranberry pomace (CP), grape pomace (GP), and Baikal skullcap (BS), subsequently incorporated either African spice (AS) or industrial marinade/pickle (IM). Results showed pH, ABTS, DPPH, FRAP, TBARS, L*a*b* color, cooking weight loss, and textural cutting force, sensory and textural profile with varying range values. Concentration increases of either CP, GP, and or BS may not always go along with ABTS, DPPH, and FRAP values, given the observed decreasing or increasing fluctuations. As oven-grilling either increased or decreased the TBARS values alongside some color and textural cutting force trends, pH variations by difference seemed more apparent at samples involving GP, before CP, and then BS. The organoleptic attributes obtained differences and resemblances from both sensory and textural profile standpoints. Overall, oven-grilling promises to moderate both physicochemical and organoleptic range values of different marinated beef entrecôte meat samples in this study.

## Introduction

The global meat production recorded about 67 million metric tonnes as of 2013, but seemingly less as of 2020 at about 60.57 million metric tonnes (McGlone, 2013; Cook, 2022; FAO, 2022, accessed September 2022). Notably, the EU as of 2016 was positioned as the third largest global beef producer by area that occupied about 11.5% (SustainBeef, 2021). Among the countries in the EU of notable interest, Poland in 2021 obtained a total national cattle population of 6.4 million by the head, which placed this country as the sixth (EU) beef producer (Nieuwsbericht, 2022). But despite this data, the level of (beef) consumption is still considered as below average (Nieuwsbericht, 2022). Among key factors that influence beef cattle (meat) quality in Poland include age, breed, diet, meat production and processing, as well as her accession to the EU in May 2004 (Domaradzki et al., 2017; Żakowska-Biemans et al., 2017). From the commercial/industrial perspective, the method by which beef is processed in Poland would follow this pathway: beef meat products > cutting plants > slaughterhouses > mechanically separated meat, and after slaughter, there would be intermediaries/outlets that direct the delivery/purchase (Szymańska, 2015).

The quality of beef meat products is very important to consumers, distributors, producers, processors, and slaughterers. Also, beef meat quality would reflect four pathways: healthiness (nutritional quality), satisfaction (organoleptic quality), security (hygienic quality), and serviceability (ease of use, ability to be processed, and prices) (Listrat et al., 2016). Besides being a great protein source for human consumption, beef meat comprises typical structural features like connective tissue, muscle fibers, and tendon, typically enriched with bioavailable iron, zinc, and selenium as well as vitamins A, B, and D (Geletu et al., 2021; Open Textbooks, 2022). At post-mortem, the accelerated glycolysis alongside the formation of lipid peroxidation products confronts beef meat, and facilitates quality deterioration (Toldrá & Flores, 2000; Martini et al., 2021). Moreover, overall beef meat value would associate with intrinsic and extrinsic cues, which consumers employ to explain their quality expectations (Domaradzki et al., 2017). When slaughtered, different beef product types do emerge largely dependent on the cut portions. When butchers cut into the bone-in rib-eyes particularly (with the bone) on each side, there would remain about six leftover boneless steaks potentially available from the beef meat. And this particularly happens between each bone-in rib-eye, which is how the entrecôte would emerge (TasteAtlas, 2023). Indeed, the making of a traditional entrecôte from the rib area of a given beef carcass certainly requires some level of specific skillset (Beef2live, 2022). Recent studies involving various quality attributes of beef carcass appear to investigate more on steak, loins, and others (Clinquart et al., 2022; Berger et al., 2018; Santos et al., 2021) much less the entrecôte.

The use of natural agents that possess preservative potentials continues to be of increasing research interest, which has been demonstrated by antioxidant and antimicrobial properties that help maintain meat quality, extend shelf-life and prevent economic loss (Al-Dalali, Li & Xu, 2022; Istrati et al., 2011). Among such natural preservative agents, marinades have been shown in recent years as increasingly applied to meat products (Cheok et al., 2011; Istrati et al., 2011; Sokołowicz et al., 2021). Dependent on the duration as well as technique of the marination process, the meat muscle can take up marinade constituents (Siroli et al., 2020). Typically requiring the immersion of meat products in a slurry/solution mix, the marination process would allow the incorporation of other edible seasonings that improve flavor development. More so, the ingredients employed in the marination process could include the likes of black/regular pepper, herbs/spices, ginger, cranberry pomace, Baikal skullcap, peanut, *etc*. (Al-Dalali, Li & Xu, 2022; Cheok et al., 2011; Istrati et al., 2011; Shahidi & Hossain, 2018; Sokołowicz et al., 2021; Martini et al., 2021; Zhang, Wu & Guo, 2016), some of which are enriched with phenols, and flavonoids (Amber et al., 2021), beneficial polyphenols (Roopchand et al., 2013), as well as antimicrobial capacities (Teplá et al., 2013).

To make beef edible, thermal processing of one form or another remains inevitable, which over the decades has advanced, from cook-chill, grilling, ohmic heating, laser-based packaging, *etc*. (Richardson, 2004; Viegas et al., 2012). Of increasing interest is grilling, which is among such thermal processes that involve temperatures above 150 °C transferred by conduction, and through direct/radiant dry heat (Schröder, 2003; Ježek et al., 2020). More so, the application of grilling of various types to meat products has been reported by several workers (Farhadian et al., 2010; Kerth, Blair–Kerth & Jones, 2003; Khan et al., 2015; Muga, Marenya & Workneh, 2021; Gómez, Ibañez & Beriain, 2019; Vişan et al., 2021). Whereas Muga, Marenya & Workneh (2021) performed modeling thin-layer drying kinetics of marinated beef submitted to infrared-assisted hot air processing, and Gómez, Ibañez & Beriain (2019) investigated the physicochemical and sensory properties of sous vide meat and meat analog products marinated and cooked at different temperature-time combinations, Vişan et al. (2021) studied the marination of Black Angus beef meat subjected to a grilling process. Other cooking methods applied to beef meat, which paved way for examination and prediction of other quality attributes (Kondjoyan et al., 2014; Onopiuk et al., 2021). Despite the published information currently available, relevant information to specific marinated oven-grilled beef entrecôte meat has not be found. Understanding the impact that combined action of marination and oven grill processes would have on such meat products as beef entrecôte is crucial from both consumer appeal and product development standpoints. To supplement existing information, this current work investigated different marinated oven-grilled beef entrecôte meat, specifically the evaluation of resultant physicochemical and organoleptic attributes. The beef entrecôte meat was provided by a reputable local bovine farm/slaughter retailer that supplies the Wroclaw’s Lower Silesia region of Poland.

## Materials and Methods

### Schematic overview of experimental program

**Figure 1:**
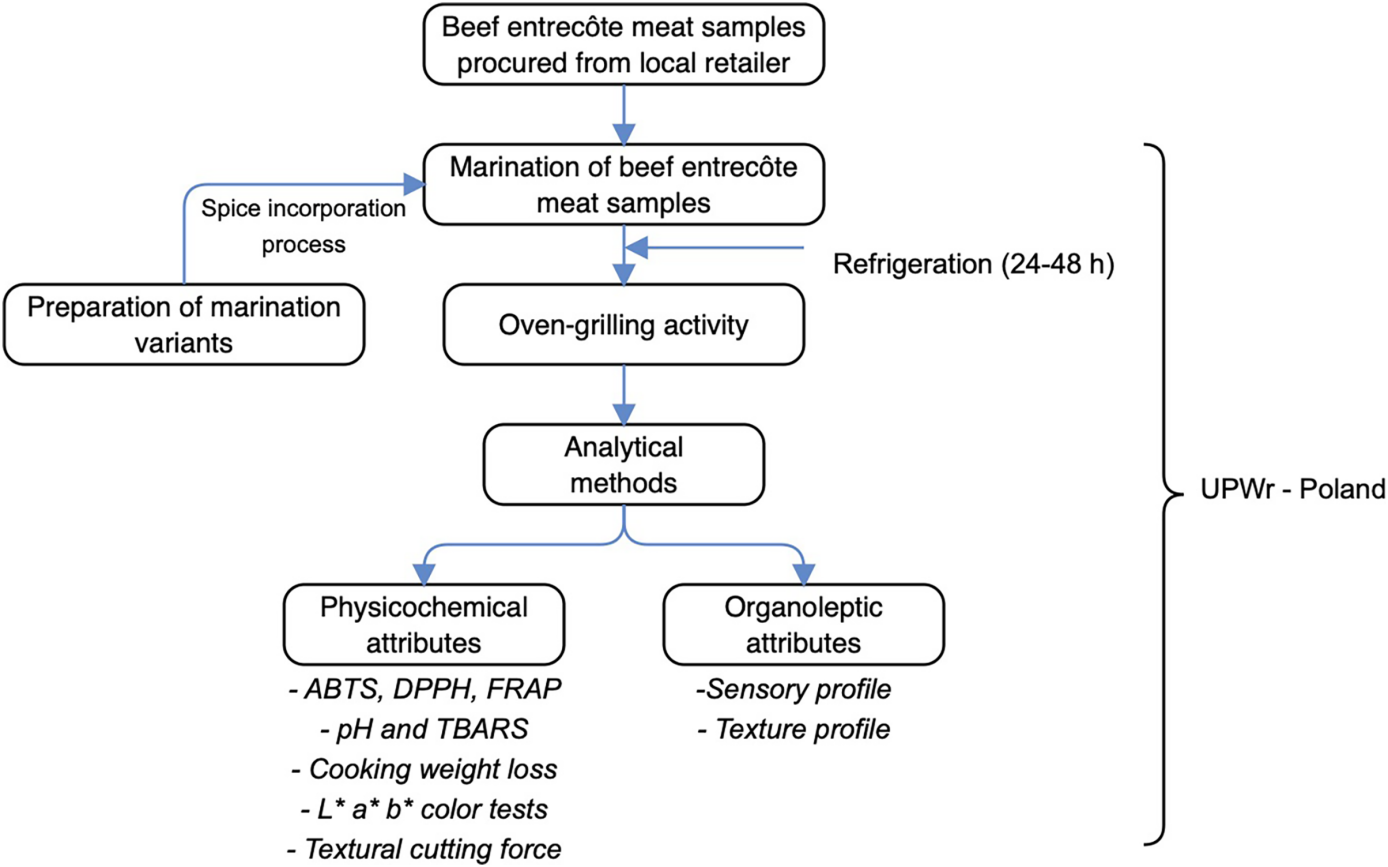
The schematic overview of the experimental program, showing the key stages, from the procurement of beef entrecôte meat samples, preparation of marinade variants, through oven-grilling activity, subsequently analytical measurements. ABTS, 2,2′-Azinobis-(3-ethylbenzthiazoline-6-sulphonate); DPPH, 2,2-diphenyl-1-picrylhydrazyl (radical scavenging activity); FRAP, ferric reducing antioxidant power; thiobarbituric acid reactive substance, TBARS; UPWr, Uniwersytet Przyrodniczy we Wrocławiu (Wroclaw University of Environmental and Life Sciences-Poland).

The schematic overview of the experimental program, demonstrating the major stages, from the procurement of beef entrecôte meat samples, preparation of marinade variants, oven-grilling activity, to analytical measurements are shown in Fig. 1. To reiterate, this current work was directed to establish how oven-grilling affected different marinated beef entrecôte meat samples specific to their physicochemical (antioxidants, pH and lipid oxidation, cooking weight loss, L*a*b* color, and textural cutting force), as well as organoleptic (sensory = flavour, appearance, tenderness, taste and flavour; texture = hardness, chewiness, gumminess, graininess, and greasiness) attributes. Added that the beef entrecôte meat has been procured from a bovine farm in Poland, the different marination variants involved cranberry pomace, grape pomace, and Baikal skullcap that subsequently incorporated African spice, and Industrial marinade/pickle. Chemicals and reagents used at this work were of analytical grade standard. Additionally, all the laboratory experimentation adhered to the relevant guidelines set out by the Department of Functional Food Product Development, Wroclaw University of Environmental and Life Sciences-Poland.

### Procurement, and preparation of beef entrecôte meat samples

Freshly processed beef entrecôte meat samples were procured from a reputable local bovine farm/slaughter retailer that supplies the Wroclaw’s Lower Silesia region of Poland. The beef entrecote meat samples (~20 kg) placed in iced packed poly-boxes were transported to the Department of Functional Food Products Development, Wroclaw University of Environmental and Life Sciences., Poland. Upon arrival, the beef entrecôte meat samples were further cut into equivalent pieces of approximate thickness (9 cm × 9 cm × 3 cm). Afterwards, all samples were subject to cold room refrigeration (~2 °C) and ready for subsequent laboratory activities of marination and oven-grilling.

### Preparation of marinades, and marination variants

The preparation of marinades followed the method described by Okpala et al. (2023). This specifically involved Baikal Skullcap (BS), cranberry pomace (CP), as well as grape pomace (GP), that subsequently incorporated constant quantities of either African spice (AS) or Industrial marinade/pickle (IM) (each constituting 4 g), alongside salt (1.6 g). For emphasis, on one hand, the African spice product (Fresh and Tasty Kebab Powder®) had been purchased from Fresh and Tasty Farms Ltd (Accra-North, Ghana), and its preparation followed the quality standards set by Food and Drugs Authority (FDA) Ghana. The label showed that this product comprised such ingredients as peanut, ginger, as well as black/regular pepper. More so, we utilized this AS product for the reason that it is increasingly being used at barbecues across Poland. On the other hand, the industrial marinade/pickle (Marinate do mięs) product had been purchased from Regis® Food Technology (Regis sp. z o.o., Kraków-Poland) and its preparation followed the quality standards set by the International Organization for Standardization (ISO), British Retail Consortium (BRC), and International Food Standard (IFS). The label showed that this product comprised such ingredients as marjoram, oregano, parsley, rosemary, and thyme. More so, we utilized this IM product for the reason that it already has an established reputation in Poland and other parts in the EU.

It is important to reiterate that the ground CP, GP, and BS served as antioxidant additives for this current study, using the method previously described (Okpala et al., 2023). The incremental concentrations of CP, GP, and BS made up 0.5%, 1%, and 1.5% by volume, which were calculated based on gram per 100 mL. Clean water served as liquid used to make up the marinade. Importantly, the marination variants were implemented as follows: (1) control where the antioxidant additive was not added (0.0%); (2) control with antioxidant additive of 0.5%; (3) control with antioxidant additive of 1.0%; (4) control with antioxidant additive of 1.5%; (5) AS incorporated with no antioxidant additive (0.0%); (6) AS incorporated with antioxidant additive of 0.5%; (7) AS incorporated with antioxidant additive of 1.0%; (8) AS incorporated with antioxidant additive of 1.5%; (9) IM incorporated with no antioxidant additive (0.0%); (10) IM incorporated with antioxidant additive of 0.5%; (11) IM incorporated with antioxidant additive of 1.0%; (12) IM incorporated with antioxidant additive of 1.5%. Following the method described by Sokołowicz et al. (2021) with modifications, the immersion method was adapted. In particular, the amount of marinade was considered adequate to completely immerse the beef entrecôte meat samples, and this applied a 1:2 ratio of weight of meat (g) and marinade volume (mL). Additionally, plastic containers approved for contact with food was used to carry out the immersion process. The beef entrecôte meat samples were dipped sufficiently in the marinade variants for 24 h at 4 °C. Subsequently, after the immersion time had completed, the marinated beef entrecote samples were then allowed to drain (5 min), and placed in folded foiled packages ready for oven-grilling activity.

### Oven-grilling procedure

The oven-grilling activity of marinated beef entrecôte meat samples employed an oven facility (CAMRY CR 6018; Serwis Centralny Camry, Warszawa, Poland). The oven-grilling operated with 2,200 W power, and set temperature of 180 °C. The beef entrecôte meat samples were placed evenly spaced in the oven-grill, which remained closed during the cooking process. Importantly, the opening of oven-grill was only when either to remove, or place new samples. Cooking time was kept constant at 5 min. During the cooking period, the internal temperature of the beef entrecote meat samples was routinely checked to ensure it was maintained roughly at about 75 °C. Upon completion of oven-grilling process, the emergent samples were allowed to cool briefly (15 min) at ambient temperature. Afterwards, emergent samples were then placed in foiled packages, submitted to refrigeration (4 °C), and then followed by analytical measurements.

### Physicochemical measurements

#### Determination of antioxidant aspects

Prior to the antioxidant tests, the preparation of meat tissue supernatant followed the method described by Bai et al. (2016) with slight modifications. This required about a gram of beef meat entrecote tissue sample subjected to homogenization at 8,000 rpm for 10 s using 9 ml of 0.9% sodium chloride buffer, briefly placed on ice, subsequently centrifuged at 4,000 rpm for 15 min at 4 °C.

The 2,2′-azinobis(3-ethylbenzothiaziline-6-sulfonate) (ABTS^+^) radical scavenging activity was performed using the method described by Bai et al. (2016) with slight modifications. The ABTS^+^ has been produced by mixing 7 mM of ABTS^+^ stock solution with 2.45 mM K_2_S_2_O_8_, subsequently incubated in darkness at 25 °C for 12–16 h. Prior to using the reagent, the ABTS^+^ solution was diluted with ethanol to an absorbance of 0.7000 ± 0.005 at 734 nm. From this, 10 μL of meat tissue supernatant were mixed with 990 μL of ABTS^+^ solution and subsequently incubated at ambient temperature of ~25 °C for 6 min. The 990 μL of ABTS^+^ solution mixed with 10 μL EtOH 70% served as the blank. Spectrophotometrically and against a blank, the absorbance was determined at 734 nm. The ABTS^+^ radical scavenging activity has been expressed by mM Trolox.

The 2,2-diphenyl-1-picrylhydrazyl (DPPH) radical scavenging activity was performed using the method described by Zhang et al. (2015) with slight modifications. Specifically, there was an already prepared DPPH solution (0.3 mM) made with ethanol. Briefly, aliquots (20 μL) from meat tissue supernatant were mixed by vortex for 1 min with 200 μL 0.3 mM of ethanolic DPPH radical solution, then allowed to stand at ambient temperature (25 °C) for 30 min in the dark. Spectrophotometrically and against a blank, the absorbance was determined at 517 nm using a UV-Vis Spectrophotometer (GENESYS™ 180; Thermo Fisher Scientific Inc., Waltham, MA, USA), and DPPH radical scavenging activity expressed in mM Trolox.

The ferric reducing antioxidant power (FRAP) measurement was performed using the method described by Lengkidworraphiphat et al. (2020) with slight modifications. This required a mixture of FRAP solution containing 10 mM 2,4,6-tripyridyl-s-triazine (TPTZ), 20 mM ferric chloride, together with 300 mM sodium acetate buffer (pH 3.6), at a ratio of 1:1:10 (v:v:v) added to the test specimen, and subsequently incubated for 30 min at 37 °C. The blank comprised 3 mL FRAP reagent mixed with 1 mL EtOH. The absorbance of resultant solution was read against a blank at 593 nm using a UV-Vis Spectrophotometer (GENESYS™ 180; Thermo Fisher Scientific Inc., Waltham, MA, USA) and FRAP value expressed as mM/dm^3^.

#### Determination of pH and lipid oxidation

The pH measurement was performed in triplicate using the method described by Barido & Lee (2022) with some modifications. This was specifically conducted before and after the oven-grilling activity. This required mixing a 5 g sample with 45 mL of distilled water in a homogenizer (PH91; SMT, Chiba, Japan) at 10,000 rpm, for 1 min using a portable pH meter (HI 99163; Hanna Instrument Company, Vöhringen, Germany) technically calibrated by buffer solutions (approximate pH 4.0, 7.0 and 9.0).

The thiobarbituric acid reactive substance (TBARS) measurement was performed in triplicate using the method described by Luciano et al. (2011) with slight modifications. This was specifically conducted before and after the oven-grilling activity. With the help of stomacher, the beef entrecôte meat samples (1.0 g) were homogenised with 10 mL of 10% trichloroacetic acid (TCA) for 1 min to precipitate proteins that are present. Subsequently, centrifugation was performed at 4,000× g (MPW-351R refrigerated; MPW Med. instruments Warszawa, Poland), and emergent mix was subject to filtration (Whatman #1 filter paper), from which 2 mL of supernatant was transferred to 2 mL of 0.06 M thiobarbituric acid. Placed in a water bath at 100 °C for 40 min, the reaction mixture was then cooled in ice-water bath (~2 min). The calibration curve was prepared using 1,1,3,3-tetra-ethoxypropane in TCA, as a standard solution. The samples were finally analysed, with absorbance was read against a blank at 532 nm using a UV-Vis Spectrophotometer (GENESYS™ 180; Thermo Fisher Scientific Inc., Waltham, MA, USA). According to the standard curve equation, TBARS values were expressed as mg of malondialdehyde (MDA) per kg of meat sample.

#### Determination of color and cooking weight loss

The color measurements were determined using the method described by Kopec et al. (2020) with slight modifications. This was specifically conducted before and after oven-grilling by way of CIE L*a*b* scale (L* = darkness; a* = redness/greenness; and b* = yellowness/blueness) using a Minolta CR-40 reflection colorimeter (Konica Minolta Sensing Europe B.V., Nieuwegein, Netherlands). Three individual measurements were taken on different areas on the beef entrecôte meat surface, and the readings display results *via* the CIE L*a*b* colorimetric system were recorded.

The cooking weight loss measurements were determined using the method described by Ali et al. (2007) with slight modifications. Specifically, the samples have been weighed prior to and after oven-grilling. The cooking weight loss depicted cooked sample (B) weight as a percentage of precooked sample (A) weight as shown by the equation below:

(1) $${\rm Cooking \;loss\; (\%) = {[(A-B)/(A)]\times 100}}$$

#### Determination of textural cutting force

The textural cutting force measurement was performed using the method described by Augustyńska-Prejsnar, Ormian & Sokołowicz (2017) with slight modifications. The specific aim was to measure the force required to cut a piece of beef entrecôte meat. The facility employed to measure cutting force (Fmax) was the Zwick/Roell machine (Zwick GmbH & Co. KG, Ulm, Germany), already equipped with Warner-Bratzler V-blade knife, which moved at a head speed of 100 mm/min and an initial force of 0.2 N. The portions of beef entrecôte meat samples were estimated cross-sectional diameter of 100 mm^2^ and 50 mm length.

### Organoleptic measurements

Organoleptic measurements of beef entrecôte meat samples comprised sensorial analysis slightly modified from Augustyńska-Prejsnar, Ormian & Sokołowicz (2018), and textural profiling slightly modified from Brambila, Bowker & Zhuang (2016). Sensory panelists constituted ten (10) staff and graduate students of the Department of Functional Food Products Development, Wrocław University of Environmental and Life Sciences (Poland), who were already familiar with the evaluation criteria set out to differentiate between the levels of the beef entrecôte meat’s flavor, appearance, tenderness, taste, and off-flavor specific to the sensorial profiling, as well as hardness, chewiness, gumminess, and graininess specific to the textural profiling. Importantly, the verbal consent taken prior to the sensory evaluation. Additionally, the panelists’ participation was voluntary, and no names/gender was reported to ensure privacy. The panelists performed the organoleptic evaluation in a well-ventilated and distraction-free environment of neutral color, and with adequate lighting. The organoleptic assessment involved the evenly cut samples already cooled to 20 ± 2 °C placed in coded white plastic plates before each panelist. Importantly, warm water was made available to each panelist to cleanse taste palates between samples. This was conducted to ensure the previous evaluation did not affect the (taste of the) new one, consistent with the work of Çakmakçı et al. (2015). The coded samples were evaluated using five-point scale (one point being the lowest score and five points being the highest) for the sensory aspects, and using 0 to 15 intensity scale for texture profile, modified from the description given by Civille & Thomas Carr (2015).

### Statistical analysis

The data, independently generated from different samples and based on a minimum of two determinations unless stated otherwise, were submitted to analysis of variance (ANOVA). Statistical significance was set at *p* < 0.05 (95% confidence level). The mean differences were resolved *post-hoc* by way of Turkey’s test. Statistica 13.0 software (StatSoft GmbH, Hamburg, Germany) was used to run the data.

## Results and discussion

### Changes in antioxidant aspects

**Figure 2:**
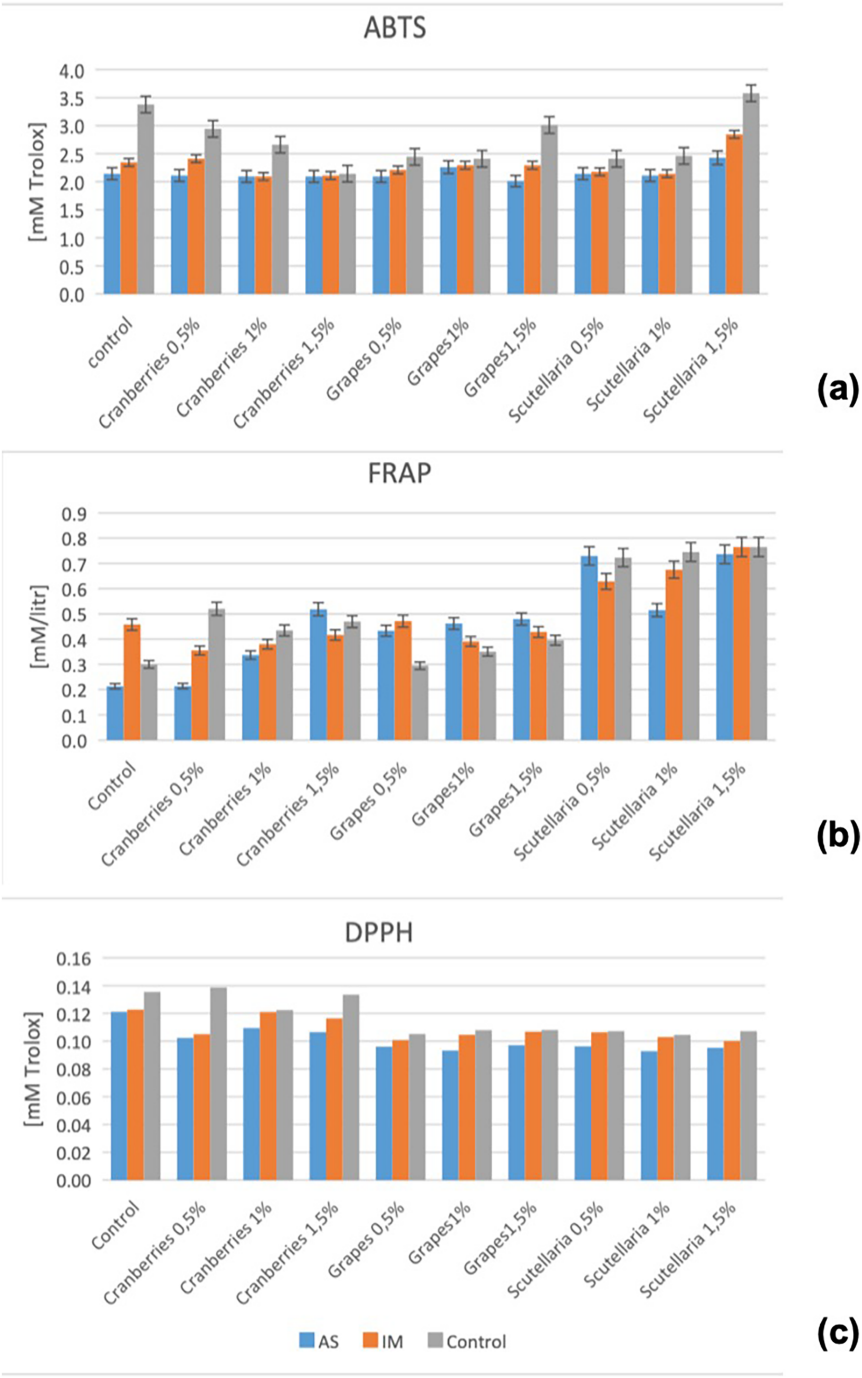
Changes in ABTS (A), FRAP (B), and DPPH (C) across the various marinated oven-grilled beef entrecôte meat samples compared to control. ABTS, 2,2′-Azinobis-(3-ethylbenzthiazoline-6-sulphonate; DPPH, 2,2-diphenyl-1-picrylhydrazyl (radical scavenging activity); FRAP, ferric reducing antioxidant power; Error bars shows mean values ± standard deviation (SD). African spice, AS; Industrial marinade/pickle, IM; BS, Baikal Skullcap. Results are expressed as mean ± standard deviation (SD).

Notwithstanding that combination of herbs and spices makes marination to produce a promising antioxidant resource, different processing methods would alter its efficacy (Thomas et al., 2010; Viegas et al., 2012). This circumstance would be particularly applicable from the point when the marinated product has just been prepared, and when it is potentially ready for consumption. In this current work, the changes in ABTS, DPPH, and FRAP values of various marinated oven-grilled beef entrecôte meat samples compared to control can be seen in Fig. 2. Across CP, GP and BS incorporating either AS or IM, different ABTS, DPPH, and FRAP ranges (for ABTS: from 2.01 ± 0.14 mM/Trolox at IM+GP 1.5% to 3.58 ± 1.89 mM/Trolox at control +BS 1.5%; for DPPH: from 0.09 ± 0.00 mM/Trolox at either AS +BS 1% or control +GP 1% to 0.14 ± 0.00 mM/Trolox at either control or IM+CP 0.5%; for FRAP: from 0.21 ± 0.01 mM/lit at control + CP 0.5% to 0.76 ± 0.00 mM/lit at either AS+BS 1.5% or IM+BS 1.5%) were found. Whereas the control samples obtained a closer DPPH range (~0.12 to 0.14 mM/Trolox), those of marinated oven-grilled beef entrecote meat samples obtained wider ranged ABTS (~2.14 to ~3.38 mM/Trolox) and FRAP (~0.21 to ~0.46 mM/lit).

Figure 2 also depicts that ABTS, DPPH and FRAP values of various marinated oven-grilled beef entrecôte meat samples specific to increasing CP, GP, and BS concentrations seems comparable with those that incorporated either AS or IM. Besides, the increasing CP, GP and or BS concentrations may not always go along with ABTS, DPPH and FRAP values given the observed decreasing or increasing fluctuations. Emphasizing the increasing CP, GP and or BS concentrations incorporating either AS or IM likens to a herb mix as strengthening the antioxidant efficacy of the marinade medium (Thomas et al., 2010; Viegas et al., 2012; Zhang et al., 2015), the heat temperatures above 120 °C resembling (oven) grilling should be capable of decreasing the antioxidant activity (Barido & Lee, 2022). It is important to understand that thermal processing like oven-grilling could open up the plant cell wall components and make them to become more sensitive so as to allow the progress of Maillard reaction (Moroney et al., 2015). Capably, the amino acids, essential oils, antioxidants, flavones, phenylethanoids, as well as sterols that are available in such herbs like BS and GP herein should scavenge, for instance, the DPPH radicals, which would help to prevent the progress of rancidity (Kim et al., 2012; Lee et al., 2014; Sáyago-Ayerdi, Brenes & Goñi, 2009).

### Changes in pH and TBARS

**Figure 3:**
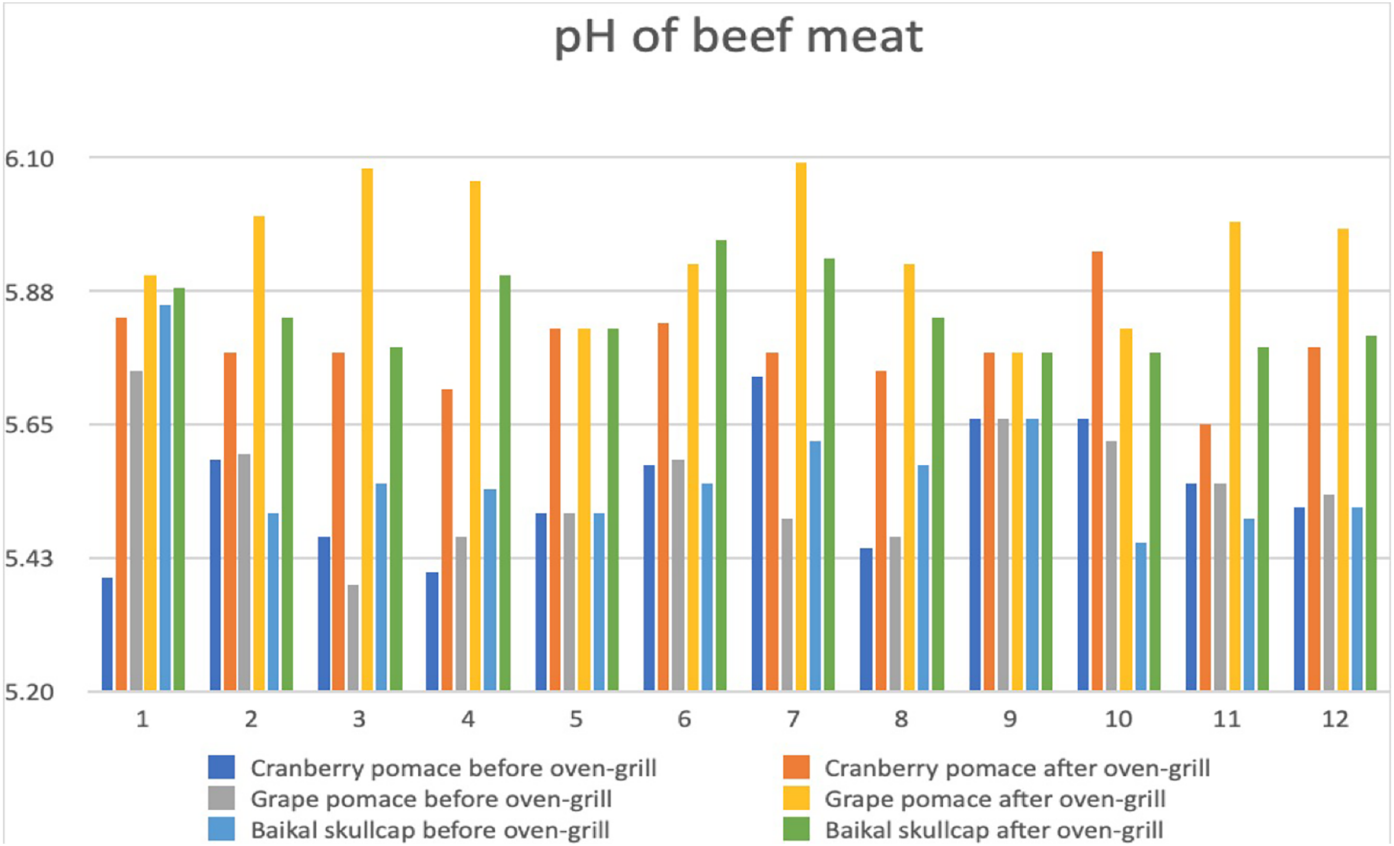
Changes in pH across the various marinated beef entrecôte meat samples before and after oven-grilling. The number representations for different colour shades are as follows: (1) control (antioxidant additive %= 0.0); (2) control (antioxidant additive %= 0.5); (3) control (antioxidant additive %= 1.0); (4) control (antioxidant additive %= 1.5); (5) AS (antioxidant additive %= 0.0); (6) AS (antioxidant additive %= 0.5); (7) AS (antioxidant additive %= 1.0); (8) AS (antioxidant additive %= 1.5); (9) IM (antioxidant additive %= 0.0); (10) IM (antioxidant additive %= 0.5); (11) IM (antioxidant additive %= 1.0); (12) IM (antioxidant additive %= 1.5). African spice, AS; Industrial marinade/pickle, IM; The antioxidant additives include cranberry pomace (CP), grape pomace (GP), and Baikal skullcap (BS).

**Figure 4:**
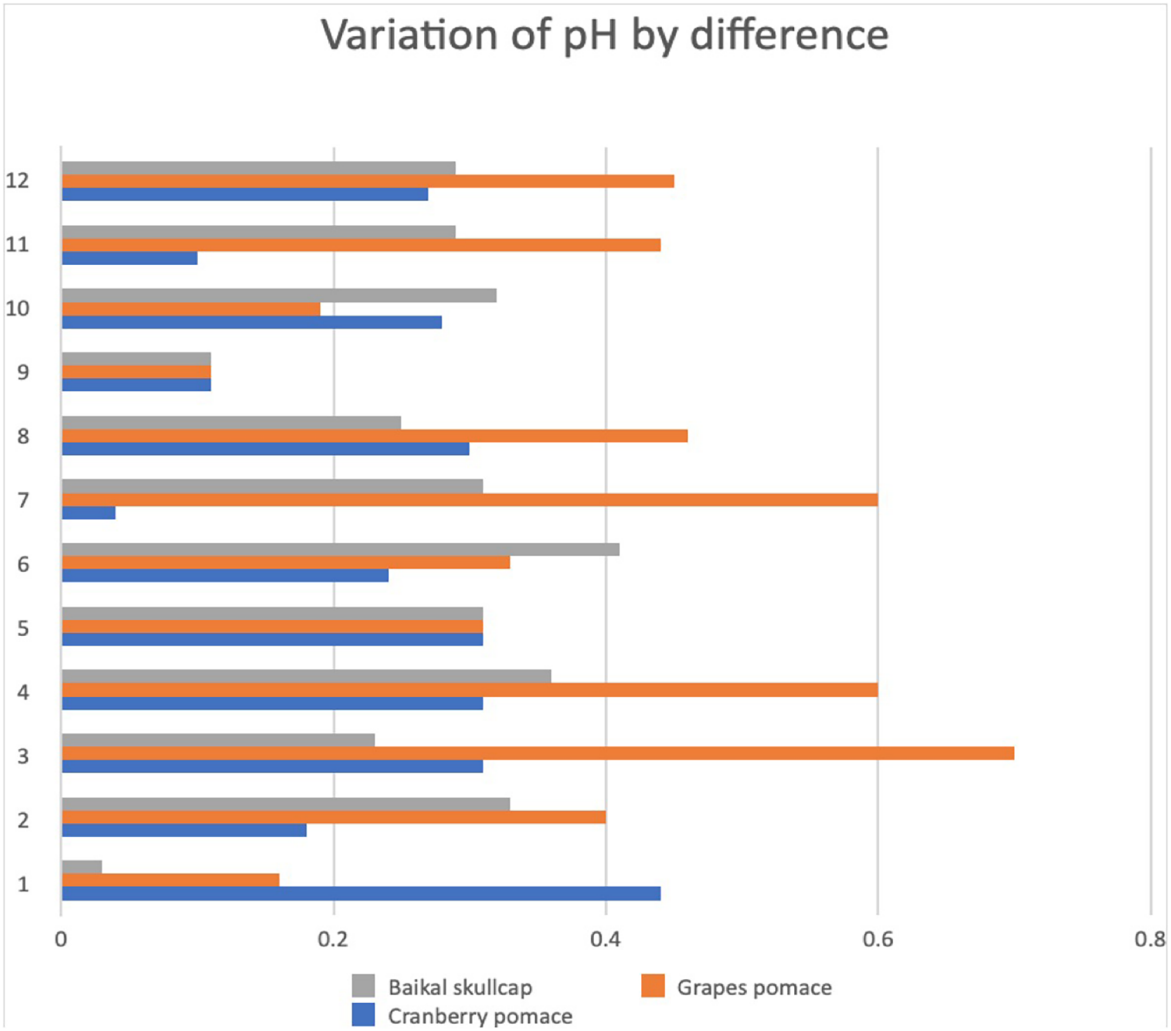
Variation of pH by difference across the various marinated oven-grilled beef entrecôte meat samples compared to control. The number representations are as follows: (1) control (antioxidant additive %= 0.0); (2) control (antioxidant additive %= 0.5); (3) control (antioxidant additive %= 1.0); (4) control (antioxidant additive %= 1.5); (5) AS (antioxidant additive %= 0.0); (6) AS (antioxidant additive %= 0.5); (7) AS (antioxidant additive %= 1.0); (8) AS (antioxidant additive %= 1.5); (9) IM (antioxidant additive %= 0.0); (10) IM (antioxidant additive %= 0.5); (11) IM (antioxidant additive %= 1.0); (12) IM (antioxidant additive %= 1.5). African spice, AS; Industrial marinade/pickle, IM; The antioxidant additives include cranberry pomace, grape pomace, and Baikal skullcap.

Applicable to beef meat quality, the changes in pH has for long been understood to associate with lower quantities of expressed juice, reflectance values, and cooking losses (Purchas, 1990). Besides pH considered as indicative of the acid concentration present, the use of marinades could eventually influence the physicochemical properties of the meat muscle (Oreskovich et al., 1992). In this current work, the changes in pH and TBARS values of the various marinated oven-grilled beef entrecôte compared to control are respectively shown in Figs. 3 and 4, as well as Table 1. Both pH and TBARS data obtained varying values. Specifically, the pH ranged from a minimum of ~5.38 at control GP before oven-grill, to a maximum of ~6.08 at control GP after oven-grill, whereas the TBARS ranged from a minimum of ~9.38 mg MDA/kg at AS+GP 0.5% to a maximum of ~26.36 mg MDA/kg at AS +GP 1%, the latter which resembled (*p* > 0.05) that of AS +GP 0.5% (~26.27 mg MDA/kg). Further, the oven-grilling seemed to either increase or decrease the TBARS values of some marinated beef entrecôte meat samples. For instance, oven-grilling appeared to noticeably reduce (*p* < 0.05) the TBARS of control (from ~17.18 to ~16.91 mg MDA/kg), in contrast to the increase when AS (from ~11.73 to 20.45 mg MDA/kg) and IM (from ~14.09 to ~24.91 mg MDA/kg) were incorporated. The detected pH and TBARS differences, which came from the application of either after oven-grilling and or together with marination variants, would most likely have shelf-life implications.

**Table 1:** Changes in thiobarbituric acid reactive substance (TBARS) across the various marinated oven-grilled beef entrecôte meat samples compared to control.

TBARS (mg malondialdehyde/kg)			Before oven-grill	After oven-grill
AS	Control		11.73^a^ ± 3.73	20.45^abc^ ± 1.16
	CP	0.5%	12.91^a^ ± 3.99	17.18^abc^ ± 4.63
		1%	23.64^a^ ± 4.11	22.91^abc^ ± 3.73
		1.5%	24.00^a^ ± 18.51	23.45^abc^ ± 9.26
	GP	0.5%	9.36^a^ ± 18.00	26.27^bc^ ± 9.51
		1%	9.73^a^ ± 0.13	26.36^c^ ± 3.21
		1.5%	23.45^a^ ± 0.39	19.82^abc^ ± 2.57
	BS	0.5%	17.27^a^ ± 14.14	16.18^abc^ ± 3.86
		1%	17.55^a^ ± 6.94	16.73^abc^ ± 2.31
		1.5%	11.64^a^ ± 6.81	13.45^abc^ ± 1.80
Control	Control		17.18^a^ ± 3.21	16.91^abc^ ± 5.91
	CP	0.5%	16.73^a^ ± 4.37	19.64^abc^ ± 0.26
		1%	12.09^a^ ± 2.44	23.00^abc^ ± 6.30
		1.5%	12.36^a^ ± 2.83	24.27^abc^ ± 8.36
	GP	0.5%	12.36^a^ ± 3.34	25.09^abc^ ± 4.89
		1%	9.82^a^ ± 0.26	19.82^abc^ ± 0.26
		1.5%	9.82^a^ ± 0.26	22.82^abc^ ± 2.96
	BS	0.5%	21.36^a^ ± 13.50	14.00^abc^ ± 2.57
		1%	9.91^a^ ± 0.90	12.45^a^ ± 0.64
		1.5%	10.36^a^ ± 1.54	13.18^abc^ ± 0.90
IM	Control		14.09^a^ ± 4.50	24.91^abc^ ± 13.11
	CP	0.5%	14.09^a^ ± 3.99	24.82^abc^ ± 13.50
		1%	16.27^a^ ± 2.19	16.36^abc^ ± 6.94
		1.5%	16.45^a^ ± 2.44	16.27^abc^ ± 7.07
	GP	0.5%	24.09^a^ ± 14.27	20.09^abc^ ± 3.73
		1%	13.73^a^ ± 5.53	16.73^abc^ ± 1.03
		1.5%	14.45^a^ ± 4.24	16.82^abc^ ± 1.41
	BS	0.5%	12.64^a^ ± 0.13	12.64^a^ ± 0.64
		1%	11.27^a^ ± 0.77	12.64^a^ ± 0.13
		1.5%	11.18^a^ ± 0.64	13.09^ab^ ± 0.26

**Note:**

Results are expressed as mean ± standard deviation (SD). Results followed by the same lowercase letter(s) do not differ significantly (*p* > 0.05). African spice, AS; Industrial marinade/pickle, IM; CP, Cranberry pomace; GP, Grape pomace; BS, Baikal Skullcap.

Figure 4 shows variation of pH by difference appears more at different marinated oven-grilled beef entrecote samples especially of GP, before CP, and then BS marination variants. Despite this, the application of oven-grill seemed to generally increase the pH regardless of marination variants. Knowing that pH value reflects the quality of beef meat and its suitability for various processing methods (Korkeala et al., 1986), to keep it reduced promises a positive shelf potential, which should avert off-odor believed to be facilitated by collagenases and other proteolytic enzymes associated with meat tenderisation (Siroli et al., 2020). To employ either increasing concentrations of CP, GP, or BS together with either AS or IM that builds up an herb mix should help to regulate the protein oxidation within the meat muscle (Xiong, 2022). Moreover, higher temperatures that come from oven-grilling should facilitate the release of oxygen, heme, and iron in meat products like beef. This situation might have made the marinated beef entrecôte meat of this current study to appear susceptible to lipid oxidation. If this situation were to progress, however, it would be demonstrated by the induced free radical production followed by undesirable off-odors/flavors (Amaral, Silva & Lannes, 2018). Largely applicable to beef carcasses, the functionality of muscles would corroborate the proportion of either slow-twitch oxidative or fast-twitch glycolytic pathways (Pereira et al., 2017).

### Changes in L*a*b* color, and cooking weight loss

**Figure 5:**
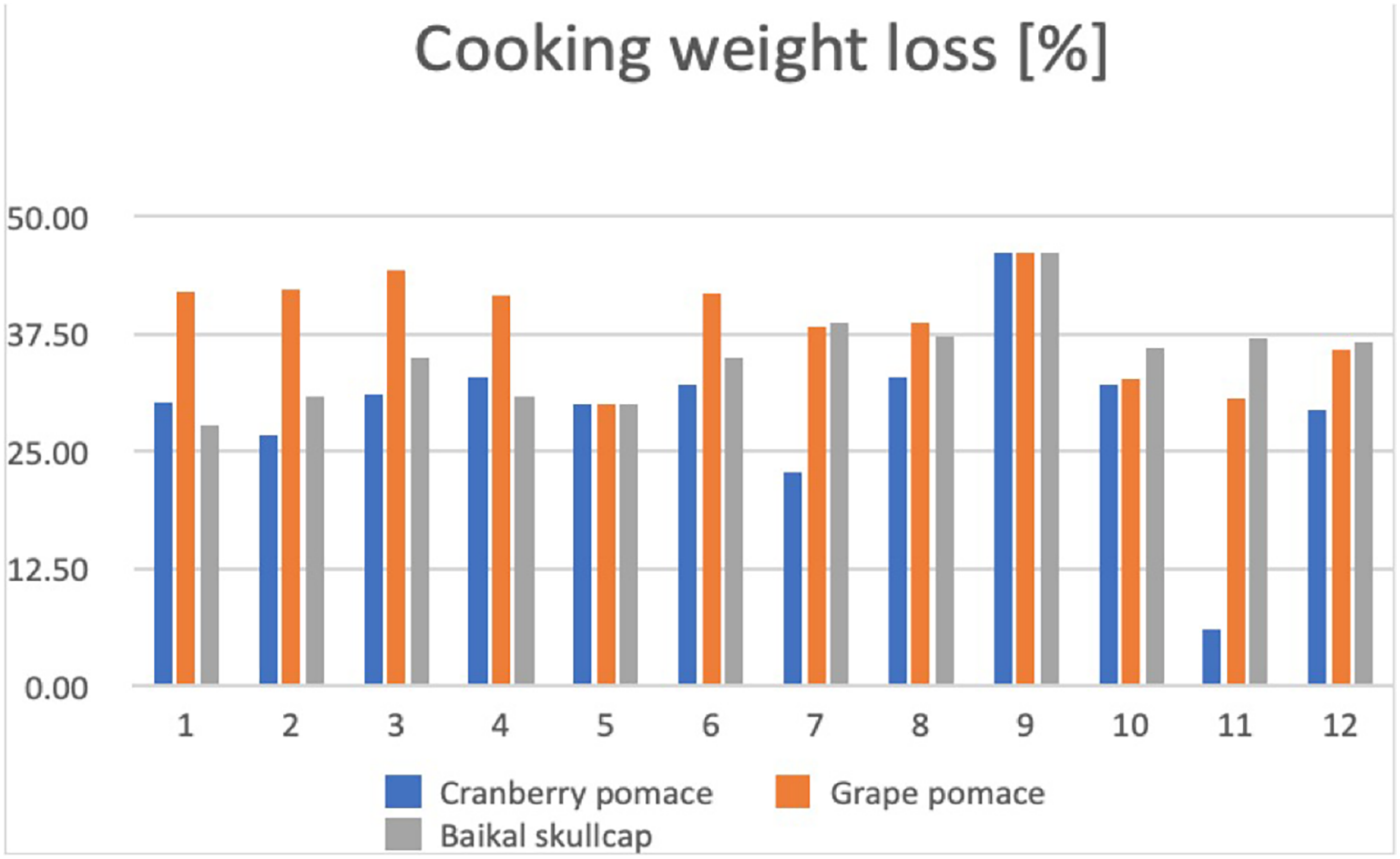
Changes in cooking weight loss (%) across the various marinated oven-grilled beef entrecôte meat samples. The number representations for different colour shades are as follows: (1) control (antioxidant additive %= 0.0); (2) control (antioxidant additive %= 0.5); (3) control (antioxidant additive %= 1.0); (4) control (antioxidant additive %= 1.5); (5) AS (antioxidant additive %= 0.0); (6) AS (antioxidant additive %= 0.5); (7) AS (antioxidant additive %= 1.0); (8) AS (antioxidant additive %= 1.5); (9) IM (antioxidant additive %= 0.0); (10) IM (antioxidant additive %= 0.5); (11) IM (antioxidant additive %= 1.0); (12) IM (antioxidant additive %= 1.5). African spice, AS; Industrial marinade/pickle, IM; The antioxidant additives include cranberry pomace, grape pomace, and Baikal skullcap.

The color stability of beef meat has been attributed to the presence of pigments, which ultimately depends on tissue composition and structure (Hashemi Gahruie et al., 2017). In this current work, the changes in L*a*b* color and cooking weight loss values of the various marinated oven-grilled beef entrecôte compared to control are respectively shown in Table 2 and Fig. 5. Varying range values of L*a*b* color (L*color: from 29.2 ± 2.4 at AS+GP 0.5% after oven-grill to 41.3 ± 1.7 at control +GP 1.0% after oven-grilling; a* color: from 2.15 ± 1.6 at IM +BS 1.5% after oven-grilling to 17.54 ± 0.82 at AS + BS 1.0%; b* color: from 3.9 ± 0.1 at control+ CP 1.0% to 15.76 ± 1.49 at IM +BS 0.5%) as well as cooking weight loss (for CP = from ~6.05% at IM with 0.5% antioxidant additive to ~46.03% at IM with no antioxidant additive; for GP = from ~29.92% at AS with no antioxidant additive to ~46.03% at IM with no antioxidant additive; for BS: from ~27.79 at control with no antioxidant additive to ~46.03% at IM with no antioxidant additive) were found. To establish a clear link when comparing color and cooking weight loss of different marinated oven-grilled beef entrecote samples seems difficult at this study. Other parameters, for instance, the pH and TBARS levels might corroborate the cooking weight loss of different marinated oven-grilled beef entrecote samples at this study. We opine this because, earlier workers like Oreskovich et al. (1992), by evaluating how marinate pH influenced texture of beef meat, understood that cooking (weight) losses could reach 45% at pH 4.24 and 5.38, but tended to decrease at pH 6.66 and 8.01.

**Table 2:** Changes in L*a*b* color of (a) cranberry pomace (CP) (b) grape pomace (GP), and (c) Baikal skullcap (BS) across the various marinated oven-grilled beef entrecôte meat samples.

(a) CP before and after oven-grill						
Sample	CP before oven-grill			CP after oven-grill		
	L	a	b	L	a	b
1	32.4^a^ ± 2.3	14.5^abc^ ± 2.3	5.3^ab^ ± 1.0	36.1^ab^ ± 3.7	6.1^abc^ ± 1.9	9.8^abc^ ± 2.8
2	31.4^a^ ± 3.0	14.1^abc^ ± 1.1	3.9^a^ ± 0.1	39.6^ab^ ± 3.2	5.4^ab^ ± 1.1	8.2^ab^ ± 1.4
3	31.8^a^ ± 1.9	15.5^bc^ ± 3.3	6.4^abc^ ± 1.7	37.8^ab^ ± 3.0	4.1^a^ ± 0.5	6.7^a^ ± 0.7
4	32.7^a^ ± 2.0	16.9^c^ ± 2.1	8.6^bcd^ ± 1.2	40.9^b^ ± 4.3	4.8^ab^ ± 0.2	8.5^abc^ ± 1.3
5	33.1^a^ ± 1.2	14.5^abc^ ± 0.4	9.3^cde^ ± 1.2	40.5^b^ ± 6.4	6.0^abc^ ± 0.9	11.1^bcd^ ± 2.7
6	32.1^a^ ± 1.3	12.9^abc^ ± 4.2	12.7^e^ ± 1.4	34^ab^ ± 3.4	5.3^ab^ ± 2.1	9.0^abc^ ± 2.1
7	32.5^a^ ± 1.1	15.8^bc^ ± 1.2	10.6^de^ ± 1.7	36.7^ab^ ± 4.5	8.0^c^ ± 1.6	11.1^bcd^ ± 1.6
8	31.8^a^ ± 0.6	12.4^ab^ ± 0.6	10.5^de^ ± 1.4	39.4^ab^ ± 3.4	5.4^ab^ ± 1.0	11.5^cd^ ± 1.1
9	34.8^a^ ± 1.4	11.1^a^ ± 1.8	7.2^abcd^ ± 2.3	40.7^b^ ± 1.2	6.4^abc^ ± 0.7	14.6^e^ ± 1.2
10	34.5^a^ ± 2.9	14.6^abc^ ± 2.4	9.6^cde^ ± 3.3	35.6^ab^ ± 4.9	4.8^ab^ ± 2.5	9.9^bc^ ± 1.8
11	32.6^a^ ± 0.9	13.7^abc^ ± 2.4	10^cde^ ± 3.9	40^ab^ ± 1.3	4.0^a^ ± 0.5	8.5^abc^ ± 0.6
12	32.3^a^ ± 2.1	14.0^abc^ ± 1.6	7.5^abcd^ ± 1.2	32.8^a^ ± 3.7	6.9^bc^ ± 1.4	13.3^de^ ± 0.5

(b) GP before and after oven-grill						
	GP before oven-grill			GP after oven-grill		
	L	a	b	L	a	b
1	33.3^a^ ± 1.6	17.0^b^ ± 2.1	10.3^bcd^ ± 1.5	35.6^abcd^ ± 1.9	4.1^abc^ ± 2.8	7.8^ab^ ± 2.5
2	33.9^a^ ± 3.4	16.0^b^ ± 1.0	5.3^a^ ± 2.1	41.3^d^ ± 1.7	3.6^ab^ ± 1.0	11.4^bcd^ ± 0.7
3	33.4^a^ ± 1.5	16.1^b^ ± 2.0	9.3^abc^ ± 1.1	34.5^abcd^ ± 6.1	3.5^a^ ± 2.2	7.1^a^ ± 1.9
4	33.6^a^ ± 4.5	11.0^a^ ± 3.2	6.3^ab^ ± 3.2	36.2^abcd^ ± 4.3	4.7^abc^ ± 0.4	10.1^abc^ ± 2.5
5	33.1^a^ ± 1.2	14.5^b^ ± 0.4	9.3^abc^ ± 1.2	40.5^cd^ ± 6.4	6.0^abc^ ± 0.9	11.1^bcd^ ± 2.7
6	33.1^a^ ± 2.7	13.4^ab^ ± 2.5	9.7^abcd^ ± 1.5	33.5^abc^ ± 4.4	3.5^a^ ± 1.5	6.5^a^ ± 2.0
7	35.3^a^ ± 1.9	14.9^b^ ± 0.7	13.5^cd^ ± 2.2	29.2^a^ ± 2.4	7.4^c^ ± 0.7	9.9^abc^ ± 1.0
8	33.3^a^ ± 1.2	15.6^b^ ± 0.4	10.1^abcd^ ± 4.0	31.3^ab^ ± 4.4	6.6^abc^ ± 2.7	9.0^ab^ ± 2.6
9	34.8^a^ ± 1.4	11.1^a^ ± 1.8	7.2^ab^ ± 2.3	40.7^cd^ ± 1.2	6.4^abc^ ± 0.7	14.6^d^ ± 1.2
10	32.7^a^ ± 1.6	15.5^b^ ± 2.3	9.8^abcd^ ± 2.0	36.9^bcd^ ± 3.1	5.4^abc^ ± 0.3	13.2^cd^ ± 0.3
11	31.2^a^ ± 1.4	15.2^b^ ± 1.0	14.4^d^ ± 2.8	29.5^ab^ ± 4.7	7.0^bc^ ± 2.3	8.6^ab^ ± 1.9
12	31.6^a^ ± 0.9	14.5^b^ ± 2.3	10.0^abcd^ ± 3.7	32.4^ab^ ± 3.2	5.2^abc^ ± 2.4	9.9^abc^ ± 3.3

(c) BS before and after oven-grill						
	BS before oven-grill			BS after oven-grill		
	L	a	b	L	a	b
1	33.7^a^ ± 5.7	16.0^cde^ ± 3.1	7.2^ab^ ± 4.3	35.1^ab^ ± 3.8	7.7^c^ ± 3.0	10.1^ab^ ± 2.4
2	34.9^a^ ± 3.9	15.1^bcde^ ± 0.8	4.3^a^ ± 0.5	31.0^a^ ± 0.5	7.3^c^ ± 0.7	10.4^ab^ ± 1.0
3	35.8^a^ ± 0.7	13.9^abcd^ ± 1.8	12.4^cde^ ± 1.5	32.2^a^ ± 5.4	5.8^abc^ ± 2.6	8.8^a^ ± 1.6
4	33.2^a^ ± 0.5	15.7^bcde^ ± 0.7	3.9^a^ ± 0.9	37.4^ab^ ± 0.9	4.2^abc^ ± 1.2	8.0^a^ ± 1.8
5	33.1^a^ ± 1.2	14.5^bcd^ ± 0.4	9.3^bc^ ± 1.2	40.5^b^ ± 6.4	6.0^bc^ ± 0.9	11.1^ab^ ± 2.7
6	34.7^a^ ± 2.3	17.5^e^ ± 0.8	11.8^cd^ ± 3.6	33.4^a^ ± 1.5	5.6^abc^ ± 2.0	10.4^ab^ ± 3.2
7	34.3^a^ ± 0.4	16.3^de^ ± 0.8	4.8^a^ ± 1.4	35.9^ab^ ± 2.0	4.4^abc^ ± 3.0	9.9^ab^ ± 3.3
8	34.4^a^ ± 3.2	13.9^abcd^ ± 1.8	11.1^c^ ± 1.2	32.3^a^ ± 2.7	4.4^abc^ ± 2.0	10.4^ab^ ± 3.2
9	34.8^a^ ± 1.4	11.1^a^ ± 1.8	7.3^ab^ ± 2.3	40.7^b^ ± 1.2	6.4^c^ ± 0.7	14.6^b^ ± 1.2
10	31.9^a^ ± 2.1	12.8^ab^ ± 0.9	11.1^c^ ± 2.2	34.9^ab^ ± 3.5	2.5^ab^ ± 1.6	9.4^a^ ± 3.2
11	34.2^a^ ± 2.7	13.2^abc^ ± 2.6	15.8^e^ ± 1.5	34.5^ab^ ± 2.3	2.3^a^ ± 1.3	9.2^a^ ± 3.6
12	34.8^a^ ± 1.7	13.6^abcd^ ± 1.2	15.3^de^ ± 1.0	35.7^ab^ ± 5.2	2.2^a^ ± 1.6	7.8^a^ ± 3.2

**Note:**

Results followed by the same lowercase letter(s) in the column of cutting force do not differ significantly (*p* > 0.05). The number representations for different color shades are as follows: (1) control (antioxidant additive %= 0.0); (2) control (antioxidant additive %= 0.5); (3) control (antioxidant additive %= 1.0); (4) control (antioxidant additive %= 1.5); (5) AS (antioxidant additive %= 0.0); (6) AS (antioxidant additive %= 0.5); (7) AS (antioxidant additive %= 1.0); (8) AS (antioxidant additive %= 1.5); (9) IM (antioxidant additive %= 0.0); (10) IM (antioxidant additive %= 0.5); (11) IM (antioxidant additive %= 1.0); (12) IM (antioxidant additive %= 1.5). African spice, AS; Industrial marinade/pickle, IM; The antioxidant additives include: (a) cranberry pomace (CP) (b) grape pomace (GP), and (c) Baikal skullcap (BS).

To a large degree, the high temperature of 180 °C and process duration of 5 min set for the oven-grilling at this current study would most likely be contributing to the cooking weight loss outcomes of the different marinated oven-grilled beef entrecote samples. Probably, this heat treatment kickstarted the fibre contractions within the intramuscular connective tissue/muscle, which might have accounted for the differences in the cooking weight loss (Ježek et al., 2020). Besides oven grilling process to bring about some decreases in cooking weight loss, the physical condition of beef entrecôte muscle might not solely depend on the degree of moisture loss influence, but would include the anticipated infiltration of the marination variants. Alongside cooking weight loss, the application of oven-grilling across CP, GP and BS probably brought about some observable color trends, depicted by either increasing or decreasing L*a*b* values. For instance, whilst the oven-grilling largely increased the L* color, and decreased the a* color, it would largely fluctuate the b* color scales of different marinated beef entrecôte meat samples. An enhanced antioxidant effect should not reflect the decreases in a* value, which would be required to stabilize the color (Libera et al., 2018). Feasibly also, the oven-grilling might have facilitated the range values of L*a*b* color as well as cooking weight to expand towards the extreme values, potentially supplemented by increment concentrations of CP, GP and BS, alongside the incorporation of AS or IM.

### Changes in textural cutting force

Defined by certain homogeneous attributes and often adapted by food processing, meat texture often instrumentally determined explains key human physiological-psychological awareness of key rheological and associated properties (Novaković & Tomašević, 2017). In this current work, the changes in textural cutting force values of the various marinated oven-grilled beef entrecôte compared to control are shown in Table 3. Incorporating either AS or IM, the textural cutting force values showed promising ranges across CP (from 35.2 ± 5.83 N at IM without antioxidant additive, to 84.7 ± 10.28 N at IM +CP 1.5%), GP (from 35.2 ± 5.83 N at IM without antioxidant additive, to 83.8 ± 21.14 N at AS +GP 0.5%) and BS (from 35.2 ± 5.83 N at IM without antioxidant additive, to 70.3 ± 27.92 N at AS + BS 1.0%) samples. Probably, the CP, GP and BS concentrations might be increasing with textural cutting force.

**Table 3:** Changes in textural cutting force across the various marinated grilled beef entrecôte meat samples compared to control.

nr	Antioxidant additive	Type of marinade	Percentage of antioxidant additive	Beef nutting force (N)
1	CP	Control	0.0	67.5^bcde^ ± 7.2
2			0.5	59.7^abcde^ ± 4.8
3			1.0	69.5^cde^ ± 11.8
4			1.5	57.6^abcde^ ± 15.9
5		AS	0.0	68.7^cde^ ± 5.2
6			0.5	35.6^a^ ± 4.0
7			1.0	37.2^ab^ ± 1.5
8			1.5	66.9^bcde^ ± 11.5
9		IM	0.0	35.2^a^ ± 5.8
10			0.5	59.2^abcde^ ± 24.4
11			1.0	81.9d^e^ ± 15.3
12			1.5	84.7^e^ ± 10.3
13	GP	Control	0.0	81.5^de^ ± 23.5
14			0.5	70.1^cde^ ± 29.0
15			1.0	59.7^abcde^ ± 10.8
16			1.5	60.9^abcde^ ± 14.9
17		AS	0.0	68.7^cde^ ± 5.2
18			0.5	83.8^de^ ± 21.1
19			1.0	62.0^abcde^ ± 8.6
20			1.5	47.9^abc^ ± 4.3
21		IM	0.0	35.2^a^ ± 5.8
22			0.5	40.6^abc^ ± 11.8
23			1.0	67.5^bcde^ ± 8.0
24			1.5	56.6^abcde^ ± 2.6
25	BS	Control	0.0	64.9^abcde^ ± 27.4
26			0.5	42.6^abc^ ± 8.6
27			1.0	66.8^bcde^ ± 10.6
28			1.5	68.0^cde^ ± 31.5
29		AS	0.0	68.7^cde^ ± 5.2
30			0.5	59.6^abcde^ ± 7.9
31			1.0	70.3^cde^ ± 27.9
32			1.5	53.5^abcd^ ± 6.2
33		IM	0.0	35.2^a^ ± 5.8
34			0.5	61.9^abcde^ ± 34.9
35			1.0	54.3^abcde^ ± 4.4
36			1.5	60.7^abcde^ ± 7.8

**Note:**

Results are expressed as mean ± standard deviation (SD). Results followed by the same lowercase letter(s) in the column of cutting force do not differ significantly (*p* > 0.05). African spice, AS; Industrial marinade/pickle, IM; CP, Cranberry pomace; GP, Grape pomace; BS, Baikal skullcap.

Earlier workers like Oreskovich et al. (1992) understood that textural properties of beef meat are more likely to change in acidic compared to alkaline conditions. In this current work, higher textural cutting force values seemed to corroborate with samples that had more acidic-like pH values. Contextualizing this observation with the composition of muscle tissue, the connective aspects like myofibrillar proteins would help to build up the meat tenderness (Migdał et al., 2020). Moreover, any increase in the cutting force could associate with the cracking phenomena, which could happen within the muscle fibers to negatively influence the muscle tenderness (Xia et al., 2012). At slaughter, the meat structure would be affected as muscle glycogen increases with resistance to stress-induced (glycogen) depletion, alongside severe pH decreases (Olsson & Pickova, 2005). Whilst (beef) entrecôte samples comprise fat, connective tissue, as well as exudative juice, the muscle mass comprise between 35–60% of animal’s total weight (Redefine Meat, 2022), all of which should be among the influential considerations that underpin the textural cutting force values of the various marinated oven-grilled beef entrecôte samples.

### Changes in organoleptic aspects

Among key organoleptic attributes, it is believed that color, flavor and texture show strong influence on consumers’ overall acceptability of meat products (Hashemi Gahruie et al., 2017). In this current work, the changes in organoleptic aspects of various marinated oven-grilled beef entrecôte samples, specifically by way of sensory and textural profiles, are shown in Tables 4 and 5. Either incorporating CP, GP and or BS, and even when involving control, AS and IM, there were minimum and maximum ranges found in sensory (Flavor: from 2.79 ± 1.22 at control+CP 0.5%, to 4.50 ± 0.79 at AS +GP 1%; Appearance: from 3.14 ± 1.35 at IM without antioxidant additive, to 4.43 ± 0.79 at AS without antioxidant additive; Tenderness: from 1.88 ± 0.90 at control + GP 0.5%, to 4.06 ± 0.84 at IM+BS 0.5%; Taste: from 2.38 ± 1.11 at control +GP 1.5%, to 4.36 ± 0.63 at AS + CP 1.0%; Off-flavor: 3.88 ± 1.35 at control +GP 1.5%, to 5.00 ± 0.00 at either control+BS 0.5%, or AS +BS 1.5%), and textural (Hardness: from 3.86 ± 1.35 at control +CP 0.5%, to 8.14 ± 1.77 at control +GP 1.5%; Chewiness: from 3.75 ± 1.57 at IM+BS 0.5%, to 8.00 ± 1.83 at control+GP 1.5%; Gumminess: from 3.71 ± 2.36 at IM+GP 0.5%, to 6.71 ± 2.06 at control+GP 0.5%; Graininess: from 2.43 ± 1.13 at IM +GP 1.5%, to 4.57 ± 2.15 at IM+CP 0.5%; Greasiness: from 2.14 ± 1.07 at IM+ GP 0.5%, to 4.63 ± 1.63 at AS +BS 0.5%) profiles.

**Table 4:** Sensory profile by way of flavour, appearance, tenderness, taste and flavour across the various marinated grilled beef entrecôte meat samples compared to control.

			Flavour	Apperance	Tenderness	Taste	Off flavor
Control	Control	0%	3.57^abcd^ ± 0.98	3.93^a^ ± 1.10	2.43^abc^ ± 1.27	3.00^abcd^ ± 1.15	4.86^ab^ ± 0.38
	CP	0.5%	2.79^a^ ± 1.22	3.43^a^ ± 1.72	3.43c^def^ ± 0.53	3.79^bcde^ ± 0.81	4.51^ab^ ± 1.12
		1%	3.79^abcd^ ± 0.57	4.29^a^ ± 0.95	2.79^abcde^ ± 0.81	3.36^abcde^ ± 1.11	4.71^ab^ ± 0.49
		1.5%	3.71^abcd^ ± 0.76	3.86^a^ ± 0.90	2.71^abcd^ ± 0.76	3.14^abcde^ ± 0.90	4.14^ab^ ± 1.46
	GP	0.5%	3.88^abcd^ ± 0.90	3.88^a^ ± 0.69	1.88^a^ ± 0.90	2.88^abc^ ± 0.90	4.63^ab^ ± 0.49
		1%	3.63^abcd^ ± 0.95	3.88^a^ ± 0.69	3.25^bcdef^ ± 0.53	3.63^bcde^ ± 0.53	4.63^ab^ ± 0.38
		1.5%	3.63^abcd^ ± 0.95	4.00^a^ ± 0.58	1.88^a^ ± 1.07	2.38^a^ ± 1.11	3.88^a^ ± 1.35
	BS	0.5%	3.31^abcd^ ± 0.45	3.75^a^ ± 0.61	3.69^def^ ± 0.96	3.44^abcde^ ± 0.70	5.00^b^ ± 0.00
		1%	3.44^abcd^ ± 0.39	3.63^a^ ± 0.79	3.38^cdef^ ± 0.91	3.52^abcde^ ± 0.70	4.88^ab^ ± 0.38
		1.5%	3.63^abcd^ ± 0.53	3.69^a^ ± 0.96	3.63^cdef^ ± 1.17	3.25^abcde^ ± 0.82	4.50^ab^ ± 1.51
AS	Control	0%	3.64^abcd^ ± 0.94	4.43^a^ ± 0.79	3.06^bcdef^ ± 1.09	3.50^abcde^ ± 1.04	4.79^ab^ ± 0.39
	CP	0.5%	3.21^abc^ ± 1.68	3.50^a^ ± 1.38	3.71^def^ ± 0.76	3.37^abcde^ ± 1.11	4.43^ab^ ± 0.98
		1%	3.50^abcd^ ± 1.19	3.29^a^ ± 1.38	3.43^cdef^ ± 0.84	4.36^e^ ± 0.63	4.86^ab^ ± 0.38
		1.5%	3.50^abcd^ ± 0.96	3.93^a^ ± 1.17	3.43^cdef^ ± 0.79	4.29^e^ ± 0.49	4.86^ab^ ± 0.38
	GP	0.5%	4.13^bcd^ ± 0.69	4.00^a^ ± 0.69	2.75^abcd^ ± 0.69	3.00^abcd^ ± 0.57	4.38^ab^ ± 0.53
		1%	4.50^d^ ± 0.79	4.13^a^ ± 0.76	2.75^abcd^ ± 1.10	4.00^cde^ ± 1.15	4.75^ab^ ± 0.38
		1.5%	3.50^abcd^ ± 1.13	3.38^a^ ± 1.13	2.88^abcdef^ ± 0.58	2.75^ab^ ± 1.25	4.50^ab^ ± 0.53
	BS	0.5%	3.57^abcd^ ± 1.03	3.50^a^ ± 1.03	3.38^cdef^ ± 1.22	3.38^abcde^ ± 0.48	4.38^ab^ ± 1.50
		1%	2.88^ab^ ± 0.79	3.19^a^ ± 0.61	3.50^cdef^ ± 0.76	3.56^abcde^ ± 0.50	4.75^ab^ ± 0.49
		1.5%	3.50^abcd^ ± 1.11	3.56^a^ ± 0.75	3.81^def^ ± 0.94	3.88^bcde^ ± 0.76	5.00^b^ ± 0.00
IM	Control	0%	3.86^abcd^ ± 1.21	3.14^a^ ± 1.35	3.36^cdef^ ± 1.38	3.5^abcde^ ± 1.19	4.36^ab^ ± 1.11
	CP	0.5%	3.43^abcd^ ± 1.27	3.71^a^ ± 1.38	2.86^abcdef^ ± 1.35	3.29^abcde^ ± 1.22	4.14^ab^ ± 1.03
		1%	3.86^abcd^ ± 0.69	3.79^a^ ± 1.35	3.00^abcdef^ ± 0.82	4.00^cde^ ± 0.65	4.79^ab^ ± 0.39
		1.5%	4.07^bcd^ ± 0.73	4.36^a^ ± 0.63	4.00^ef^ ± 0.65	4.21^de^ ± 0.81	4.86^ab^ ± 0.38
	GP	0.5%	4.25^cd^ ± 1.11	3.75^a^ ± 1.07	3.75^def^ ± 1.07	3.88^bcde^ ± 1.00	4.75^ab^ ± 0.38
		1%	3.88^abcd^ ± 0.90	4.06^a^ ± 0.58	3.00^abcdef^ ± 0.90	2.94^abc^ ± 1.21	4.00^ab^ ± 1.53
		1.5%	4.06^bcd^ ± 0.73	4.00^a^ ± 0.82	3.13^bcdef^ ± 1.11	4.00^cde^ ± 0.82	4.25^ab^ ± 1.11
	BS	0.5%	3.50^abcd^ ± 1.38	3.38^a^ ± 1.07	4.06^f^ ± 0.84	3.44^abcde^ ± 1.29	4.75^ab^ ± 0.38
		1%	3.81^abcd^ ± 0.63	4.00^a^ ± 0.90	3.56^cdef^ ± 0.50	3.94^bcde^ ± 0.91	4.50^ab^ ± 1.13
		1.5%	4.13^bcd^ ± 1.41	3.31^a^ ± 0.81	3.88^def^ ± 1.11	3.63^bcde^ ± 1.43	4.75^ab^ ± 0.38

Note:

Results are expressed as mean ± standard deviation (SD). Results followed by the same lowercase letter(s) do not differ significantly (*p* > 0.05). African spice, AS; Industrial marinade/pickle, IM; Cranberry pomace, CP; Grape pomace, GP; Baikal skullcap, BS.

**Table 5:** Textural profile by way of hardness, chewiness, gumminess, graininess, and greasiness across the various marinated grilled beef entrecôte meat samples compared to control.

			Hardness	Chewiness	Gumminess	Graininess	Greasiness
Control	Control	0%	5.86^bcdefg^ ± 1.57	6.00^abcdefgh^ ± 0.82	5.57^abc^ ± 0.53	4.00^a^ ± 1.41	3.00^abc^ ± 1.41
	CP	0.5%	3.86^ab^ ± 1.35	5.14^abcdef^ ± 1.57	4.43^abc^ ± 1.51	3.00^a^ ± 2.00	2.86^abc^ ± 1.21
		1%	7.00^fgh^ ± 1.63	7.00^fgh^ ± 1.15	5.14^abc^ ± 1.57	3.29^a^ ± 1.98	2.29^ab^ ± 1.11
		1.5%	6.71^efgh^ ± 1.11	6.29^cdefgh^ ± 1.25	4.71^abc^ ± 1.70	3.43^a^ ± 1.72	3.29^abc^ ± 2.43
	GP	0.5%	7.57^gh^ ± 1.62	7.86^gh^ ± 1.68	6.71^c^ ± 2.06	2.57^a^ ± 2.07	2.86^abc^ ± 1.77
		1%	6.43^defgh^ ± 1.27	5.86^abcdefgh^ ± 1.07	4.57^abc^ ± 2.23	3.43^a^ ± 1.90	3.14^abc^ ± 1.95
		1.5%	8.14^h^ ± 1.77	8.00^h^ ± 1.83	6.14^abc^ ± 2.61	2.57^a^ ± 2.30	3.43^abc^ ± 2.37
	BS	0.5%	4.63^abcde^ ± 2.45	5.13^abcdef^ ± 1.72	4.38^abc^ ± 2.51	2.63^a^ ± 1.57	4.25^abc^ ± 1.57
		1%	4.63^abcde^ ± 1.70	5.25^abcdef^ ± 1.38	4.63^abc^ ± 1.99	3.00^a^ ± 2.36	4.44^abc^ ± 1.84
		1.5%	4.50^abcde^ ± 1.11	5.00^abcdef^ ± 1.89	5.25^abc^ ± 1.41	2.75^a^ ± 2.24	4.13^abc^ ± 1.27
AS	Control	0%	5.71^abcdefg^ ± 2.43	5.71^abcdefg^ ± 2.43	4.71^abc^ ± 2.56	3.29^a^ ± 1.80	2.71^ab^ ± 1.38
	CP	0.5%	4.29^abcd^ ± 1.80	4.43^abcd^ ± 2.15	4.15^abc^ ± 2.12	3.86^a^ ± 2.27	3.86^abc^ ± 1.86
		1%	4.14^abc^ ± 1.35	5.14^abcdef^ ± 1.95	4.00^ab^ ± 1.53	3.43^a^ ± 1.51	3.29^abc^ ± 1.70
		1.5%	5.71^abcdefg^ ± 1.38	6.00^abcdefgh^ ± 1.73	5.00^abc^ ± 2.16	4.43^a^ ± 1.40	2.57^ab^ ± 1.27
	GP	0.5%	6.50^defgh^ ± 2.10	5.43^abcdef^ ± 2.07	5.00^abc^ ± 1.73	2.57^a^ ± 1.27	3.14^abc^ ± 2.41
		1%	6.71^efgh^ ± 1.38	6.00^abcdefgh^ ± 1.41	6.14^abc^ ± 1.95	2.43^a^ ± 1.62	2.71^ab^ ± 1.80
		1.5%	5.29^abcdef^ ± 1.98	6.00^abcdefgh^ ± 1.83	6.43^bc^ ± 2.88	3.00^a^ ± 1.63	2.86^abc^ ± 2.12
	BS	0.5%	5.38^abcdefg^ ± 2.43	4.88^abcdef^ ± 2.37	5.50^abc^ ± 2.14	3.38^a^ ± 2.82	4.63^bc^ ± 1.63
		1%	4.63^abcde^ ± 1.50	4.88^abcdef^ ± 1.27	3.75^a^ ± 1.38	3.50^a^ ± 2.07	4.50^abc^ ± 1.98
		1.5%	4.63^abcde^ ± 1.63	4.25^abc^ ± 1.98	4^ab^ ± 2.36	2.88^a^ ± 2.12	4.38^abc^ ± 1.29
IM	CP	0%	4.57^abcde^ ± 1.72	4.86^abcdef^ ± 2.12	3.86^ab^ ± 2.12	3.43^a^ ± 1.72	3.00^abc^ ± 1.63
		0.5%	6.29^cdefgh^ ± 2.06	6.57^defgh^ ± 1.62	6.00^abc^ ± 1.63	4.57^a^ ± 2.15	3.43^abc^ ± 2.23
		1%	6.29^cdefgh^ ± 1.25	6.57^cdefgh^ ± 1.40	6.00^abc^ ± 1.91	3.86^a^ ± 1.57	4.00^abc^ ± 2.31
		1.5%	5.43^abcdefg^ ± 2.15	5.43^abcdef^ ± 2.23	5.00^abc^ ± 2.52	3.71^a^ ± 2.21	2.71^ab^ ± 1.38
	GP	0.5%	4.43^abcd^ ± 1.27	4.57^abcde^ ± 1.81	3.71^a^ ± 2.36	2.57^a^ ± 1.51	2.14^a^ ± 1.07
		1%	5.86^bcdefg^ ± 2.19	5.86^abcdefgh^ ± 2.41	6.00^abc^ ± 2.45	2.86^a^ ± 1.46	3.29^abc^ ± 1.60
		1.5%	5.86^bcdefg^ ± 2.67	5.29^abcdef^ ± 2.63	5.43^abc^ ± 2.70	2.43^a^ ± 1.13	2.86^abc^ ± 1.07
	BS	0.5%	3.50^a^ ± 1.46	3.75^a^ ± 1.57	4.13^abc^ ± 1.70	3.63^a^ ± 2.36	4.13^abc^ ± 1.60
		1%	5.00^abcdef^ ± 1.00	4.44^abcd^ ± 1.27	4.19^abc^ ± 1.68	3.38^a^ ± 1.98	4.38^abc^ ± 1.46
		1.5%	4.00^ab^ ± 1.27	3.88^ab^ ± 1.40	4.13^abc^ ± 1.46	4.25^a^ ± 3.10	3.75^abc^ ± 0.95

Note:

Results are expressed as mean ± standard deviation (SD). Results followed by the same lowercase letter(s) do not differ significantly (*p* > 0.05). African spice, AS; Industrial marinade/pickle, IM; Cranberry pomace, CP; Grape pomace, GP; Baikal skullcap, BS.

The organoleptic attributes obtained some statistical differences (*p* < 0.05) as well as resemblances (*p* > 0.05) from sensory and textural standpoints. Specifically, such resemblances might suggest the panelists were unable to differentiate between some specific samples at this study. The fluctuating values would also suggest the increasing the CP, GP and BS concentrations appears not always going along with some of the sensorial and textural profile attributes. Interestingly, the panelists provided somewhat consistently higher off-flavour scores to most evaluated marinated oven-grilled beef entrecôte samples. The application of marinades is believed to have the ability to influence color of meat, yet not negatively when sensorially evaluated by panelists (Siroli et al., 2020). From the combination of instrumental texture and sensory tenderness acceptability, however, it should be possible to detect when beef meat toughness becomes unacceptable (Schilling et al., 2003). More so, differences in flavor, juiciness, and tenderness detectable by organoleptic evaluation may well suggest the preservative potential of marinades (Kim et al., 2010).

## Conclusion

Different marinated oven-grilled beef entrecôte meat, specifically the resultant physicochemical and organoleptic attributes were investigated. Varying range values in pH, ABTS, DPPH, FRAP, TBARS, L*a*b* color scales, cooking weight loss, and textural cutting force, sensory and textural profile were detected. Moreover, the oven-grilling applied across CP, GP and BS largely produced some major color trends, which was either to increase or decrease the L*a*b* values. Also, increasing the CP, GP and BS concentrations might be help to increase the textural cutting force compared to control. The statistical differences and resemblances at organoleptic attributes were demonstrated by varying ranges, from sensory and textural profile standpoints. Considering that antioxidant values fell below control at some instances, the marinated oven-grilled beef entrecote samples of this work should have shelf promise. This has to be verified using different refrigerated packaging and storage conditions, during which the evaluation of other quality attributes like microbiological, volatile amines, amino acid as well as fatty acid/flavor profiles could be ascertained.

## Supplemental Information

10.7717/peerj.15116/supp-1 Supplemental Information 1 Raw data.

## Additional Information and Declarations

### Data Availability

The following information was supplied regarding data availability: The raw data are available in the Supplemental File.
